# EFhd2 Affects Tau Liquid–Liquid Phase Separation

**DOI:** 10.3389/fnins.2019.00845

**Published:** 2019-08-13

**Authors:** Irving E. Vega, Andrew Umstead, Nicholas M. Kanaan

**Affiliations:** ^1^Department of Translational Science and Molecular Medicine, College of Human Medicine, Grand Rapids, MI, United States; ^2^Neuroscience Program, Michigan State University, Grand Rapids, MI, United States; ^3^Department of Neurology, University of Michigan, Ann Arbor, MI, United States; ^4^Hauenstein Neuroscience Center, Mercy Health Saint Mary’s, Grand Rapids, MI, United States

**Keywords:** EFhd2, tau, liquid–liquid phase separation, neurodegeneration, tauopathy, Alzheimer’s disease

## Abstract

The transition of tau proteins from its soluble physiological conformation to the pathological aggregate forms found in Alzheimer’s disease and related dementias, is poorly understood. Therefore, understanding the process that modulates the formation of toxic tau oligomers and their conversion to putative neuroprotective neurofibrillary tangles will lead to better therapeutic strategies. We previously identified that EFhd2 is associated with aggregated tau species in AD brains and the coiled-coil domain in EFhd2 mediates the interaction with tau. To further characterize the association between EFhd2 and tau, we examined whether EFhd2 could affect the liquid–liquid phase separation properties of tau under molecular crowding conditions. We demonstrate that EFhd2 alters tau liquid phase behavior in a calcium and coiled-coil domain dependent manner. Co-incubation of EFhd2 and tau in the absence of calcium leads to the formation of solid-like structures containing both proteins, while in the presence of calcium these two proteins phase separate together into liquid droplets. EFhd2’s coiled-coil domain is necessary to alter tau’s liquid phase separation, indicating that protein–protein interaction is required. The results demonstrate that EFhd2 affects the liquid–liquid phase separation of tau proteins *in vitro*, suggesting that EFhd2 modulates the structural dynamics of tau proteins.

## Introduction

Proteinopathies are a group of diseases whose pathological hallmark is aberrant accumulation of protein aggregates (Dugger and Dickson, 2017; Sweeney et al., 2017). The molecular processes leading to the transition from physiological to pathological protein conformations is a research area of significant interest. The microtubule-associated protein tau undergoes transitions from soluble monomer to oligomer and subsequently into paired helical filaments that comprise the neurofibrillary tangles (NFTs), a pathological hallmark in Alzheimer’s disease (AD) and other neurodegenerative disorders (Lee et al., 2001; Guo et al., 2017; Polanco et al., 2017). The molecular mechanisms involved in the formation and accumulation of tau aggregates is poorly understood. Several studies indicate that tau oligomers are a toxic form, while NFTs may represent a neuroprotective form due to sequestration of the more toxic tau species (Alonso et al., 2006; Vega et al., 2008; Kuchibhotla et al., 2014; Goedert and Spillantini, 2017). Understanding how these two thermodynamically different tau structures are formed and coexist is crucial to deciphering the molecular mechanisms involved in tau-mediated neurodegeneration.

**FIGURE 1:**
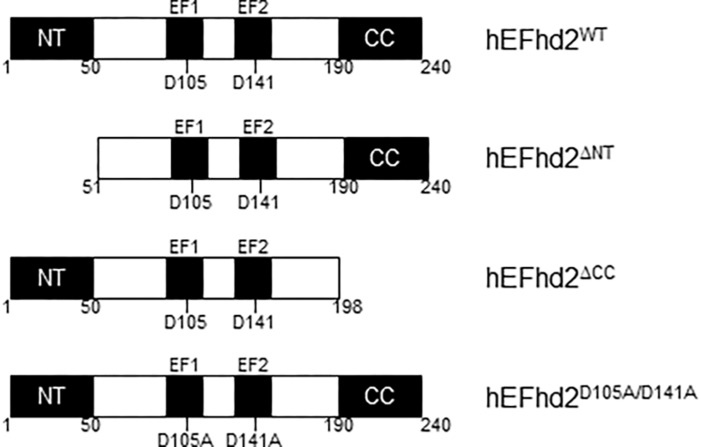
Human EFhd2 protein. Illustrated representation of recombinant human proteins used in this study. EFhd2 is a 240 amino acids protein that has two EF-hand motifs that expand from amino acids 96–114 (EF1) and 132–160 (EF2). As part ate residues at the EF-loops mediate calcium binding. At the N-terminus (NT) has a poly-Alanine motif and a coiled-coil domain (CC) at the C-terminus. The illustration is not at scale.

We previously identified EFhd2 as a tau-associated protein in a tauopathy mouse model (JNPL3) and AD brains (Vega et al., 2008; Ferrer-Acosta et al., 2013a). EFhd2 is highly expressed in the central nervous system in comparison to other tissues but the function of neuronal EFhd2 is poorly understood (Vega et al., 2008). EFhd2 mouse and human protein sequences are 93% identical (Ferrer-Acosta et al., 2013b). EFhd2 is a 240 amino acid protein with two structural units mediating protein–protein interactions, a polyalanine motif at the N-terminus and a coiled-coil domain at the C-terminus (Figure 1). EFhd2 has two functional EF-hand motifs, demonstrating that EFhd2 is a calcium binding protein (Vega et al., 2008; Ferrer-Acosta et al., 2013b). Cdk5, a kinase associated with the pathophysiology of AD, phosphorylates EFhd2 at serine 74, significantly reducing its calcium binding activity (Vázquez-Rosa et al., 2014). Further characterization of the EFhd2-tau association demonstrated that EFhd2 colocalized with pathological tau in the somatodendric compartment in AD and this association is increasingly detected as neurodegeneration progresses in JNPL3 mice (Vega et al., 2008; Ferrer-Acosta et al., 2013a). EFhd2 co-purified with sarkosyl insoluble tau (Vega et al., 2008; Ferrer-Acosta et al., 2013a). Immuno-gold labeling of the sarkosyl insoluble tau fraction from AD temporal lobe demonstrated that EFhd2 and tau coexist in filamentous structures and electron microscopy analysis confirmed that EFhd2 self-associates forming filamentous structures *in vitro* (Ferrer-Acosta et al., 2013a). The presence of calcium reduces the formation of these structures and the coiled-coil domain is required for EFhd2 self-association (Ferrer-Acosta et al., 2013a). Deletion of EFhd2’s coiled-coil domain also affected its associations with tau proteins, while deletion of the N-terminus had no effect (Ferrer-Acosta et al., 2013a). Recently, we demonstrated that incubation of EFhd2 with tau’s three repeat domain promote the formation of amyloid structures as detected by increased Thioflavin S signal (Vega et al., 2018). However, it is unclear whether EFhd2 can modulate the formation of tau aggregates.

The physiological and pathological role of intracellular phase separation is starting to be appreciated as the formation of functional and dynamic entities within the cellular milieu (Aguzzi and Altmeyer, 2016; Banani et al., 2017). The ability of intrinsically disordered proteins to demix into liquid droplets or form solid-like structures suggests that mechanisms that regulate these transitions could demark the boundaries between health and diseased states (Aguzzi and Altmeyer, 2016; Banani et al., 2017). Recent studies showed that tau proteins can undergo liquid phase condensation *in vitro*, forming distinct and dynamic liquid droplets under molecular crowding conditions (Ambadipudi et al., 2017; Hernández-Vega et al., 2017; Shin and Brangwynne, 2017). Tau can facilitate tubulin polymerization, driving the nucleation of microtubules, within these liquid droplets (Ambadipudi et al., 2017). Thus, tau proteins preserve biological function within liquid droplets, suggesting that tau physiological and pathological functions could be mediated within distinct intracellular protein condensate states. Here, we demonstrate that EFhd2 forms solid-like and liquid droplets structures, which are mediated by its coiled-coil domain. We also show that EFhd2 modulates liquid–liquid phase separation behavior of tau, demonstrating, for the first time, that EFhd2 directly converts tau liquid phase behavior to solid-like structures *in vitro* and this phenomenon is controlled by calcium.

## Materials and Methods

### EFHD2 Gene Cloning

Human EFHD2 (Accession # Q96C19) wild type gene, N-terminal and C-terminal truncated and D105A/D141A mutants were produced by custom gene synthesis (Integrated DNA Technologies). The synthesized genes were subcloned into a bacterial expression vector pp-80L (5Prime Cat No. 2400850) in frame with an N-terminal 6x-Histidine tag, between *Bam*HI (5′ site) and *Hin*dIII (3′ site) restriction sites. After ligation, the plasmids were transformed into One Shot^TM^ BL21 (DE3) *E. coli* chemically competent cells (Thermo Fisher Scientific, Cat No. # C600003). Colonies were checked for protein expression. Plasmid DNA was extracted from colonies with positive expression of the expected protein molecular weight and subjected to DNA sequencing (GENEWIZ). Protein bands were also excised and subjected to trypsin in-gel digestion and tandem mass spectrometry analysis to confirm protein sequence (Supplementary Figure 1).

### Recombinant Protein

For recombinant EFhd2 protein purification, a 50 mL LB/Ampicillin (50μ g/mL) pre-culture was incubated overnight at 37°C with constant shaking. 300 mL LB/Ampicillin was inoculated to 0.2 OD_600 *rmnm*_ using the saturated pre-culture and incubated at 37°C with constant shaking for 1 h or until the culture reach 0.5–0.7 OD_600 nm_. Then, IPTG was added to the culture to a final concentration of 0.5 mM. The culture was incubated for 3 h at 37°C. The culture was centrifuge at 10,000 rpm for 10 min at 4°C. The bacteria pellet was resuspended in 10 mL of Lysate Buffer [1x PBS with 5 mM Imidazole (pH 8.0)] and lysed by sonication on ice. The resulting lysate was centrifuged at 14,000 rpm for 10 min at 4°C. The supernatant was incubated with 500μ L of HIS-SELECT (Sigma, Cat No. # H0537-25 mL) beads (pre-calibrated in Lysate Buffer) and incubated overnight at 4°C with constant rotation. The beads were allowed to settle by gravity and the supernatant was removed. The beads were resuspended in 10 mL of Lysate buffer and transferred to an empty 10 mL column. The Lysate buffer was allowed to flow through until reaching the top of the beads bed and the recombinant protein was eluted with 500μ L of 1x PBS containing 250 mM Imidazole (pH 8.0). Four 500μ L fractions were collected and checked on SDS-PAGE. Those fractions containing recombinant protein were pooled and buffer exchanged to 1x PBS pH 8.0, using centricon spin filters 3 kDa cutoff. The final volume was 250μ L and concentration was kept below 3μ g/μL to prevent EFhd2 spontaneous self-aggregation. GFP tagged (Tau-GFP) and untagged (Tau) recombinant tau (Accession # P10636-8) proteins with C-terminal 6x poly-histidine tags were produced and purified as previously described (Combs et al., 2017). Briefly, T7 express *E. coli* cells were induced to express proteins with IPTG, followed by collection of cells and lysis of the cell pellet. Proteins were extracted through a three-step chromatography process with a GE Akta Pure system. First, proteins were purified on a Talon metal affinity resin column, the resulting proteins were further cleaned using size exclusion chromatography with an S500 column, and finally the samples were further polished over an anion exchange column. DTT (1 mM) was added to the proteins stocks, which were aliquoted and stored at −80°C until used for experiments.

### Recombinant Protein Labeling and Quantification

The recombinant proteins were labeled using Alexa Fluor^®^ 594 Protein Labeling Kit (Molecular Probe Cat No. # A10238) or FluoReporter^TM^ FITC Protein Labeling Kit (Molecular Probe Cat No. # F6436) following the manufacture instructions. Briefly, purified recombinant protein was buffer exchanged into 1x PBS. Sodium Bicarbonate was added to final concentration of 100 mM (1M stock solution). Then, 100 μL of purified recombinant protein was added to a vial of reactive dye. The labeling reaction was stirred for 1 h at room temperature. The labeled protein was purified using the provided resin bed and centrifugation at 1100 × *g* for 3 min. A protein series dilution and BSA as concentration standard (1, 2, 4, 8, 16 μg/μL) were resolved on SDS-PAGE and stained with Coomassie (R250). The distained gel was imaged in a GelDoc (BioRad) and the intensity of the stained protein bands was determined using the quantity tools of Image Lab software (Version 5.2.1 build 11).

### Liquid–Liquid Phase Separation

**FIGURE 2:**
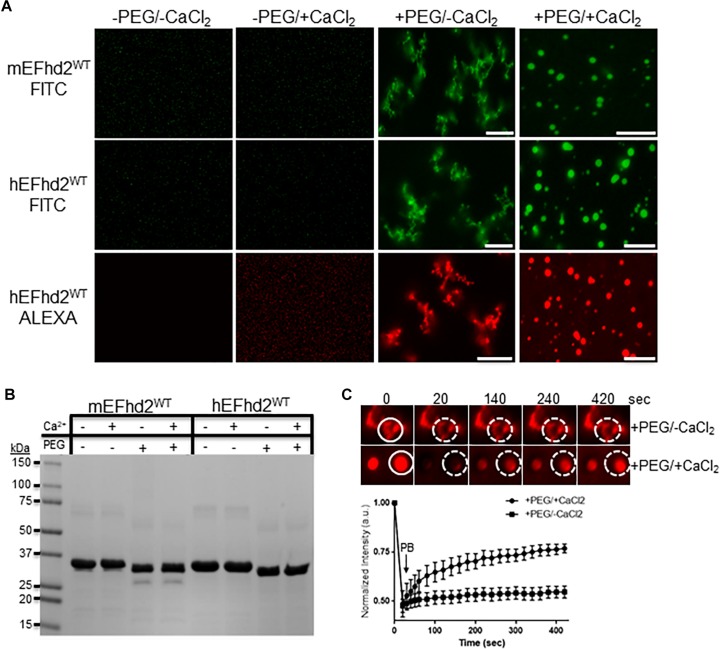
EFhd2 condense into distinct high-order structures. **(A)** Mouse (mEFhd2) and human (hEFhd2) EFhd2 orthologous were labeled with fluorescence dyes (FITC or ALEXA). The labeled proteins (16 gμM) were subjected to liquid–liquid phase separation in absence (–PEG) or presence (+PEG) of 10% Polyethylene glycol (PEG) as crowding agent plus or minus 10 mM CaCl_2_ and visualized by confocal microscopy. **(B)** Representative Coomassie stained protein gel of mEFhd2 and hEFhd2 proteins used. **(C)** Human EFhd2 wild type protein (16 μM) in the presence or absence of 10 mM CaCl_2_ was subjected to fluorescence recovery after photobleaching (FRAP). The graph shows fluorescence recovery with time in seconds (sec) after photo-bleaching (PB). The intensity was normalized to the first time point before photo-bleaching and shown as the mean and standard error of the mean (SEM), *n* = 8. The white circles illustrate the photobleached (PB) area in the sample without CaCl_2_- The images are representative of, at least, three independent experiments. Scale bar = 10 μm.

A pre-determined concentration of labeled recombinant protein was diluted in 40μ L of reaction buffer containing 10% Polyethylene glycol (PEG) 3000 monodisperse solution, 10 mM HEPES (pH7.6), 150 mM NaCl, 5 mM DTT, and 0.1 mM EGTA. EGTA was not added when CaCl_2_ was used in the reaction. The proteins were visualized using a confocal microscope at 60x with oil immersion (Nikon Confocal Microscope A1). All images were collected within 1 h after the samples were prepared. After each experiment, the samples were collected and 10 μL of the protein solution was resolved in SDS-PAGE and visualized by Coomassie staining. The presence of PEG induces a noticeable mobility shift on the migration of EFhd2 proteins in SDS-PAGE (Figure 2B). A mixture of recombinant Tau-GFP (2μ M) and Tau (2μ M) was used to avoid signal saturation. The experiments were repeated, at least, three times and independently validated in a different lab from recombinant protein purification to liquid phase separation.

### Fluorescence Recovery After Photobleaching (FRAP) Analysis

EFhd2 samples were prepared as described in the Liquid–liquid phase separation section. Only samples with crowding agent (PEG 3000) plus or minus 10 mM CaCl_2_ were subject to FRAP analysis. Briefly, at 60x magnification and 6x optical zooming, EFhd2 solid-like and liquid droplets were outlined with a circular overlay object and assigned as “stimulation” (bleaching) or “reference” (unbleached) (∼8 per field of view). Similar number of circular objects were placed on areas without either of the structures and assigned as “background.” This process was repeated in, at least, three different view fields, per sample. The FRAP parameters were as follows: a single pre-bleaching image (T0), bleaching at laser power set at 100 and 1 frame/2 s rate, an immediate post-bleach image, and several images over a total of 7 min. The FRAP curves were calculated by normalizing fluorescence signal to the background and reference signals was determined using these functions in the Nikon Elements Software. The extracted data was normalized to the intensity of the first image before photobleaching and analyzed in GraphPad Prism 6. The values were graph as mean and standard error of the mean. All imaging was done with the same acquisition settings (i.e., scan speed, resolution, magnification, optical zoom, gain, offset, and laser intensity).

## Results

### EFhd2 Proteins Condense Into Distinct High-Order Structures

Previous studies showed that EFhd2 self-associates and forms amyloid structures in a concentration dependent manner and independent of nucleation factors such as heparin (Ferrer-Acosta et al., 2013a). Recently it was shown that tau, FUS, TDP3, and other amyloid proteins form liquid droplets under molecular crowding conditions, suggesting that these proteins may maintain different intracellular liquid phase states (Ambadipudi et al., 2017; Banani et al., 2017; Hernández-Vega et al., 2017; Shin and Brangwynne, 2017; Zhang et al., 2017). To determine if EFhd2 also undergoes liquid–liquid phase separation, both recombinant mouse and human EFhd2 protein orthologs were purified and labeled with fluorescent dyes. Labeled proteins were subjected to different conditions to induce liquid–liquid phase separation similar to that reported for tau and other proteins. In our experiments, PEG 3000 was used as a molecular crowding agent and the reaction buffer contained 150 mM NaCl. Recombinant EFhd2 protein was used at much lower concentrations (4–16 μM) than previously used to report its self-association (30–40 μM) (Ferrer-Acosta et al., 2013a).

The results demonstrate that EFhd2 condenses into different high order structures in response to specific chemical environments (Figure 2). In the absence of molecular crowding, EFhd2 did not undergo liquid–liquid phase separation (Figure 2A; −PEG/-CaCl_2_ and −PEG/ + CaCl_2_). In contrast, both mouse and human EFhd2 orthologs formed amorphous structures under molecular crowding (Figure 2A; +PEG/-CaCl_2_) that resemble solid-like structures observed with other intrinsically disordered proteins (Patel et al., 2015; Zhang et al., 2017; Wegmann et al., 2018). In the presence of 10 mM calcium chloride (CaCl_2_), EFhd2 formed liquid droplets like those formed by tau and other intrinsically disordered proteins. EFhd2 condensation is not due to the presence of the fluorescent tag, since EFhd2 labeled with two different fluorescent tags show the same protein dynamic in presence and absence of calcium (Figure 2A) and unlabeled EFhd2 proteins formed these same structures as observed using differential interference contrast in the transmitted light channel of the confocal microscope system (Supplementary Figure 2). Both mouse and human EFhd2 protein orthologs showed the same condensation behavior, indicating that EFhd2 condensation capacity is conserved. After confocal imaging, the protein samples were resolved in SDS-PAGE and visualized by Coomassie staining (Figure 2B). As previously reported, PEG affected the mobility of recombinant proteins in SDS-PAGE (Odom et al., 1997). This effect is due to changes in SDS-PAGE that affects the electrophoretic mobility of proteins that run behind the PEG front (Odom et al., 1997). Human EFhd2 protein was used in all subsequent experiments.

Previous studies, using fluorescence recovery after photobleaching (FRAP), showed that proteins within liquid droplets are highly dynamic, whereas proteins within solid-like structures are arrested in glassy solid or amyloid fiber states (Patel et al., 2015; Ambadipudi et al., 2017; Banani et al., 2017; Hernández-Vega et al., 2017; Riback et al., 2017; Shin and Brangwynne, 2017; Zhang et al., 2017; Wegmann et al., 2018). FRAP analyses were performed to determine the dynamic behavior of EFhd2 within the two condensate states (Figure 2C). EFhd2 liquid-droplets’ fluorescence signal recovered ∼50% after photobleaching (Figure 2C). However, EFhd2’s solid-like structures, formed in the absence of calcium, did not show FRAP. These results confirmed the dynamic nature of EFhd2’s droplets formed in the presence of calcium and the static structure of the solid-like state in absence of calcium. Additionally, the results suggest that calcium enhances EFhd2 protein dynamics.

### Mutation of EFhd2’s Calcium Binding Sites Prevents Liquid-Droplet Formation

We conducted several controls experiments to study the effect that calcium has on EFhd2 protein dynamics. To determine if NaCl contributes, in combination with CaCl_2_, to EFhd2 condensation, experiments were performed in the absence of 150 mM NaCl and under molecular crowding conditions (Supplementary Figure 3). Even with the absence of 150 mM NaCl, EFhd2 formed solid-like and liquid droplet structures in absence or presence of CaCl_2_, respectively, under crowding conditions (Supplementary Figure 3). The same results were obtained in experiments containing 2 mM NaCl in the presence and absence of CaCl_2_ (data not shown). These results demonstrate that NaCl does not play a role in EFhd2 condensation. We also assessed whether other divalent ions, such as Mg^2+^, could contribute to EFhd2 changes in protein dynamic (Supplementary Figure 4). Recombinant EFhd2 incubated in different concentrations of MgCl_2_ did not form liquid droplets under molecular crowding conditions (Supplementary Figure 4). These results indicate that calcium ions, and neither chloride nor other divalent ions, play an important role in EFhd2 protein dynamics. Additionally, electrostatic interaction in aqueous salt solutions does not drive the observed structures.

**FIGURE 3:**
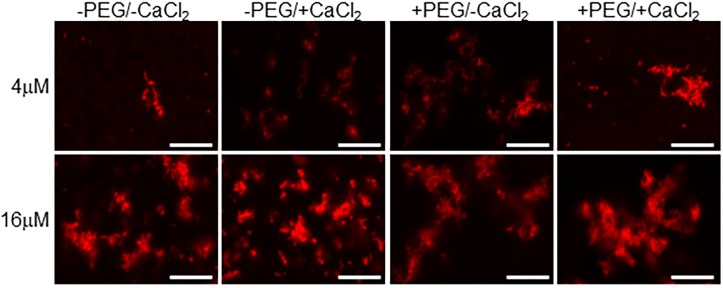
Calcium binding drives EFhd2 liquid-droplet formation. Human EFhd2 D105A/DH41A mutant was subjected to liquid–liquid phase separation in absence [–PEG) or presence [+PEG) of 10% Polyethylene glycol (PEG) as crowding agent plus or minus 10 mM CaCl_2_- The proteins were visualized by confocal microscopy. The images are representative of, at least, three independent experiments. Scale bar = 10 μm.

To determine if the integrity of EFhd2’s calcium binding sites are required for the formation of liquid-droplets, we used human EFhd2 protein bearing a missense mutation of conserved aspartate residues (D105A and D141A) within each EF-hand motif (Figure 1). We and others previously demonstrated that mutating these aspartate residues to alanine render EFhd2 unable to bind calcium (Ferrer-Acosta et al., 2013a; Park et al., 2016). hEFhd2^D105A/D^^141^^A^ formed solid-like structures in the absence and presence of crowding agent (Figure 3). The addition of calcium did not promote the formation of liquid droplets (Figure 3). These results indicate that mutation of EFhd2’s calcium binding sites affects the formation of EFhd2 liquid droplet but not solid-like structures. Importantly, since all EFhd2 recombinant proteins used are 6x His-tagged, this experiment also helps rule out the effect that 6x His-tagging could exert on the formation of EFhd2 liquid droplets. The result indicates that calcium and not the 6x His-tagged is what promotes the formation of EFhd2 liquid droplets.

### EFhd2’s Coiled-Coil Domain Is Required for Liquid–Liquid Phase Separation

**FIGURE 4:**
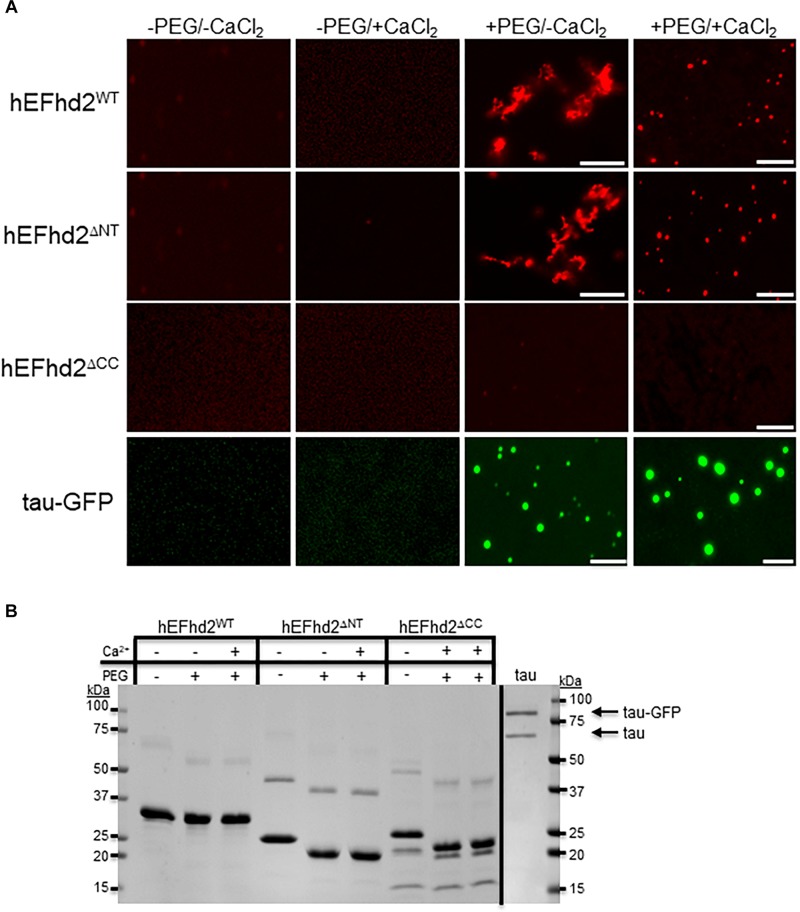
Coiled-coil domain is required for EFhd2 liquid–liquid phase separation. **(A)** Wild type human EFhd2 (hEFhd2^WT^), N-terminal (hEFhd2^Δ*NT*^), and coiled-coil (hEFhd2^Δ*CC*^) truncated mutants ALEXA-labeled recombinant proteins (4 μM) were subjected to liquid–liquid phase separation in absence (–PEG) or presence (+PEG) of 10% Polyethylene glycol (PEG) as crowding agent plus or minus 10 mM CaCl_2_-Recombinant tau (4 μM) was subjected to the same conditions as EFhd2. **(B)** EFhd2 wild type (WT), N-terminal (ΔNT), and coiled-coil (ΔCC) truncation mutant proteins and Tau proteins were resolved in SDS-PAGED and visualized by Coomassie blue staining. The images are representative of, at least, three independent experiments. Scale bar = 10 μm.

Previously, we demonstrated that deletion of EFhd2’s coiled-coil domain affected its dimerization and association with tau proteins, while deletion of the N-terminus did not affect these protein–protein interactions (Ferrer-Acosta et al., 2013a). To demonstrate that EFhd2 condensation behavior is due to intermolecular interaction and not solely to the presence of the crowding agent, we investigated the role that the N-terminus and coiled-coil domain play in liquid–liquid phase separation (Figure 4). The EFhd2 N-terminal truncation mutant (hEFhd2^ΔNT^) formed solid-like structures when calcium was absent and liquid droplets when calcium was present under molecular crowding conditions, similar to the structures observed with wild type EFhd2 (hEFhd2^WT^; Figure 4). The results indicate that the N-terminus is not required for the formation of EFhd2’s solid-like structures or liquid droplets (Figure 4). In contrast, deletion of the C-terminus coiled-coil domain (hEFhd2^ΔCC^) prevented EFhd2 condensation (Figure 4). hEFhd2^ΔCC^ protein did not undergo liquid–liquid phase separation in the absence or presence of crowding agent and calcium, indicating that while calcium changes structural dynamics, the coiled-coil domain is required for EFhd2 phase condensation. These results indicate that EFhd2 liquid condensation is mediated by protein–protein interaction. Under the same conditions and protein concentration used for EFhd2, recombinant tau formed liquid droplets similar to those previously reported in the presence or absence of CaCl_2_ (Figure 4) (Patel et al., 2015; Ambadipudi et al., 2017; Hernández-Vega et al., 2017; Riback et al., 2017; Zhang et al., 2017; Wegmann et al., 2018). Thus, calcium does not affect tau liquid phase condensation as it does for EFhd2 (Figure 4).

### EFhd2 Protein Affects Tau Liquid–Liquid Phase Separation

**FIGURE 5:**
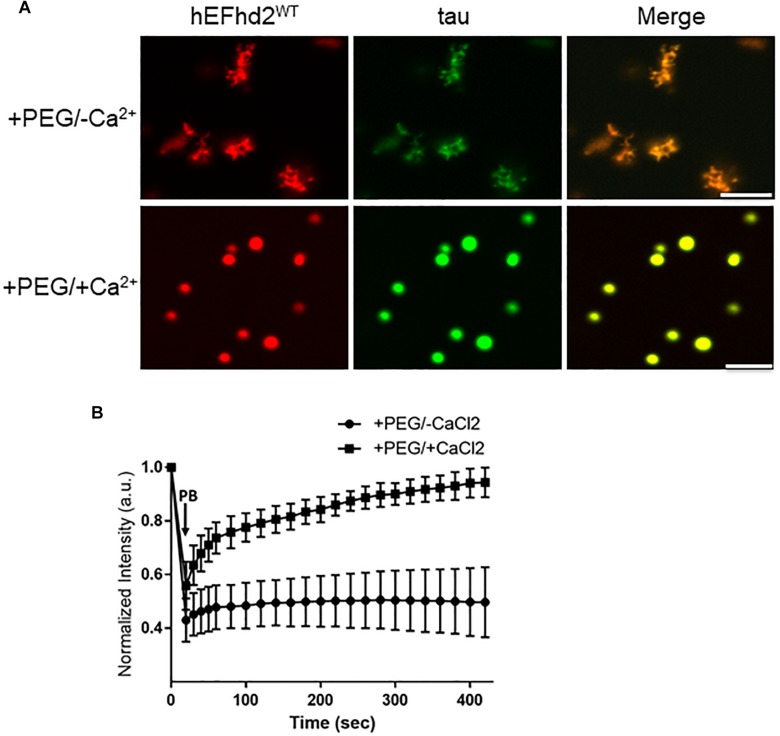
EFhd2 affects tau liquid–liquid phase separation. **(A)** Wild type human EFhd2 (hEFhd2^WT^) recombinant protein (4 μM) and equimolar concentration of recombinant tau (4 μM) were subjected to liquid–liquid phase separation in absence (–PEG; not shown) or presence (+PEG) of 10% Polyethylene glycol (PEG) as crowding agent plus or minus 10 mM CaCl_2_- The images are representative of, at least, three independent experiments. Scale bar = 10 μm. **(B)** Co-incubated EFhd2 wild type protein (4 μM) and tau (4 μM) in the presence or absence of 10 mM CaCl_2_ was subjected to FRAP. The graph shows fluorescence recovery with time in seconds (sec) after photo-bleaching (PB). The intensity was normalized to the first time point before photo-bleaching and shown as the mean and standard error of the mean (SEM) through time, *n* = 11.

The effect that EFhd2 has on tau proteins and its role in tauopathy is still unknown. Since both EFhd2 and tau undergo liquid–liquid phase separation, we assessed whether EFhd2 could influence tau liquid phase separation. Recombinant EFhd2 (red) and tau (green) proteins were combined at equimolar concentration in conditions that induced liquid–liquid phase separation (Figure 5). As expected, EFhd2 and tau did not demix in the absence of the crowding agent (Supplementary Figure 5). In contrast, EFhd2 and tau co-localized in solid-like structures in the presence of the crowding agent and absence of calcium (Figure 5A; +PEG/-Ca^2+^). As shown on Figure 3, tau alone forms liquid droplets while EFhd2 forms solid-like structures under this condition. Thus, this result indicates that EFhd2 changes tau proteins liquid phase separation to solid-like structures. Conversely, EFhd2 and tau come together into liquid droplets in the presence of calcium and crowding agent (Figure 5A; +PEG/ + Ca^2+^). To further assess the effect that EFhd2 has on tau liquid–liquid phase separation, we conducted FRAP analyses of co-incubated EFhd2 and tau in presence and absence of calcium under molecular crowding conditions (Figure 5B). The results showed that solid-like structures did not recover after photobleaching, confirming that these structures formed by EFhd2 and tau are not dynamic (Figure 5B). In contrast, EFhd2-tau droplets formed in the presence of calcium showed recovery after photobleaching, indicating that calcium influences the molecular dynamics of EFhd2-tau structures formed under molecular crowding conditions.

**FIGURE 6:**
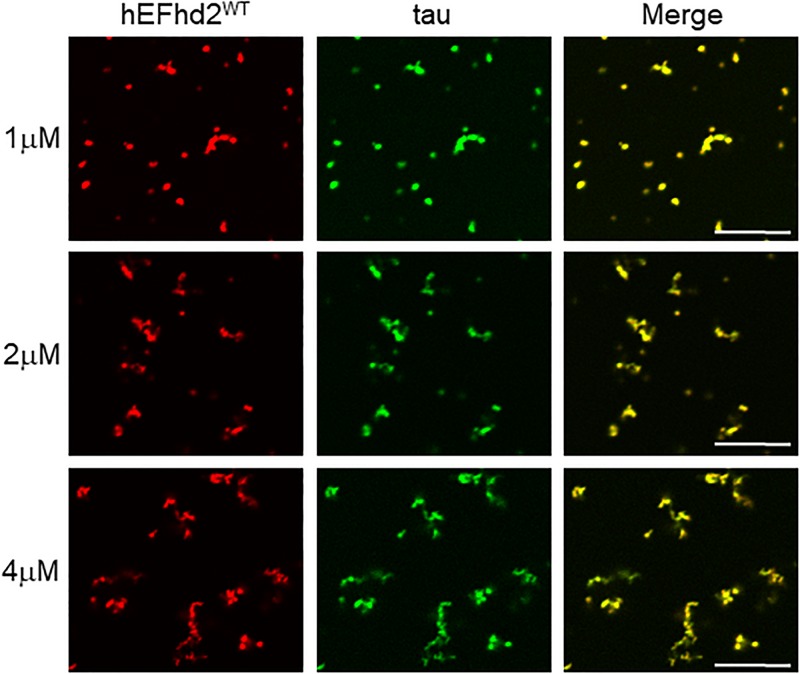
EFhd2 submolar concentrations promotes the formation of tau solid-like structures in absence of calcium. Wild type human EFhd2 (hEFhd2^WT^) recombinant protein at 1, 2, and 4 μM concentrations with recombinant tau (4 μM) were subjected to liquid–liquid phase separation in presence of 10% Polyethylene glycol (PEG) as crowding agent without CaCl_2_- The images are representative of, at least, three independent experiments. Scale bar = 10 μm.

To determine if EFhd2 concentration plays a role in its effect on tau liquid–liquid phase separation, submolar concentrations of recombinant EFhd2 were incubated with 4 μM of recombinant tau in absence of CaCl_2_ under molecular crowding conditions (Figure 6). In the absence of calcium, EFhd2 at 1 and 2 μM promoted the formation of solid-like structures containing both EFhd2 and tau proteins (Figure 6). No tau droplets were observed despite the lower concentration of EFhd2, indicating that submolar EFhd2 concentrations can affect tau dynamics. These results indicate that EFhd2 modulates tau condensate phase behavior *in vitro*.

**FIGURE 7:**
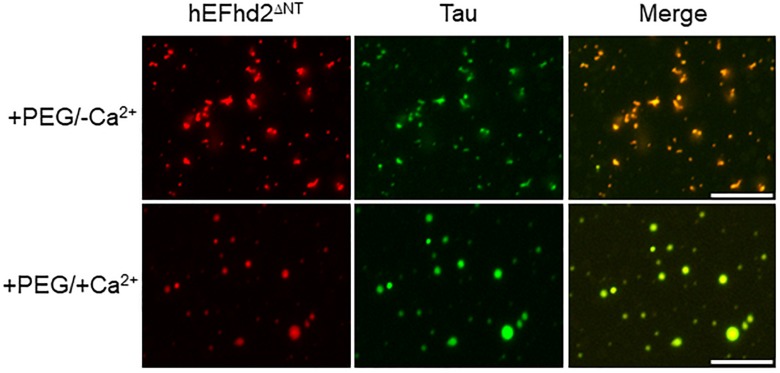
EFhd2 N-terminus is not required for affecting Tau liquid phase condensation, human EFhd2 NT-terminal truncation mutant (hEFhd2^ΔNT^) protein (4 μM) and equimolar concentration of recombinant Tau (4 μM) were subjected to liquid–liquid phase separation in presence (+PEG) of 10% Polyethylene glycol (PEG) as crowding agent plus or minus 10 mM CaCl_2_- The images are representative of, at least, three independent experiments.

Deletion of EFhd2 N-terminus does not affect its association with tau proteins (Ferrer-Acosta et al., 2013a). Thus, we investigated whether EFhd2 N-terminal truncation mutant (EFhd2^ΔNT^) could affect tau liquid phase behavior as shown with wild type EFhd2 (Figure 7). EFhd2^ΔNT^ and tau co-localized in amyloid structures in the presence of crowding agent without calcium (Figure 6; +PEG/-Ca^2+^) and in liquid droplets when incubated with crowding agent and calcium (Figure 6; +PEG/ + Ca^2+^). These results indicate that EFhd2’s N-terminus is not required to affect tau liquid phase behavior *in vitro*.

**FIGURE 8:**
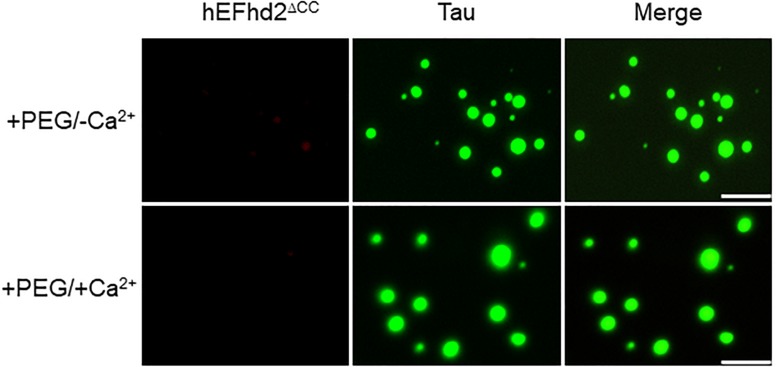
EFhd2 coiled-coil domain is required for affecting Tau liquid phase condensation, human EFhd2 coiled-coil deletion mutant (hEFhd2^ΔCC^) protein (4 μM) and equimolar concentration of recombinant Tau (4 μM) were subjected to liquid–liquid phase separation in presence (+PEG) of 10% Polyethylene glycol (PEG) as crowding agent plus or minus 10 mM CaCl_2_- The images are representative of, at least, three independent experiments. Scale bar = 10 μm.

Previously, we showed that EFhd2’s coiled-coil domain is required for its association with tau proteins (Ferrer-Acosta et al., 2013a). Therefore, we investigated the role of EFhd2’s coiled-coil domain in affecting tau liquid phase behavior (Figure 8). As shown in Figure 3, EFhd2 coiled-coil domain deletion mutant (EFhd2^Δ*CC*^) did not form detectable liquid-droplets or solid-like structures. Moreover, EFhd2^Δ*CC*^ did not affect tau liquid phase separation in the presence of crowding agent and calcium (Figure 8). This result indicates that EFhd2’s coiled-coil domain is required to affect tau’s liquid phase behavior, suggesting that protein–protein interactions drive EFhd2 modulation of tau condensate state.

## Discussion

EFhd2 condensate phase behavior ranges from solid-like to liquid droplets structures under crowding conditions. In the absence of calcium, EFhd2 is arrested in solid-like structures that are not dynamic, while calcium induces the formation of dynamic EFhd2 liquid droplets. These results indicate that the concentration of calcium and/or regulatory signals that modulate EFhd2’s calcium binding capacity can have an impact on EFhd2 protein dynamics. The coiled-coil domain is required to form solid-like and liquid droplet structures, indicating that EFhd2 protein–protein interaction drives the formation of these structures while calcium promotes enhanced EFhd2 protein dynamics. These changes in protein dynamics may have consequences on EFhd2 physiology. For example, EFhd2 serves as a scaffold for the function of Syk, SLP-65, and PLCγ2 downstream of the activation of the B-cell receptor, inducing calcium influx (Kroczek et al., 2010). EFhd2 has also facilitates F-actin remodeling necessary for migration and invasion of cancer cells (Huh et al., 2013, 2015; Park et al., 2016). Although the role of calcium signaling dysregulation in cancer cells is still poorly understood, increases in calcium levels could be the driving force to facilitate the effect of EFhd2 on nucleation and polymerization of actin filaments.

We also show that EFhd2 affects tau’s condensate phase behavior. EFhd2, in the absence of calcium, promotes tau to co-localize in solid-like structures. In the presence of calcium, however, EFhd2 and tau phase separate together into liquid droplets. The effect that EFhd2 has on tau condensate phase behaviors requires EFhd2’s coiled-coil domain, indicating that EFhd2 interaction with tau proteins affects tau condensate phase behavior.

The role that EFhd2 plays in neurodegeneration is still unclear (Vega, 2016). The association of EFhd2 and tau in AD, identification of EFhd2 and tau in filamentous structures, their colocalization in the somatodendritic compartment and copurification in the sarkosyl-insoluble fraction indicates that EFhd2 is associated with pathological tau in AD (Vega et al., 2008; Ferrer-Acosta et al., 2013a). We previously showed that Cdk5 phosphorylation of EFhd2 affects its calcium binding capacity (Vázquez-Rosa et al., 2014). Hyperactivation of Cdk5 in AD brain is associated with phosphorylation of tau proteins, leading to its dissociation from microtubules and formation of oligomers (Wilkaniec et al., 2016). Taken together, hyperactivated Cdk5 could phosphorylate EFhd2, affecting its calcium binding activity and, consequently, its protein dynamics. The accumulation of EFhd2 in solid-like state could then drive tau aggregation into oligomers and/or NFTs. Further studies are required to test this working hypothesis.

## Conclusion

The data provide further evidence that EFhd2 shares molecular and biochemical characteristics with known intrinsically disordered proteins associated with neurodegenerative diseases. This suggests that EFhd2 may play an important role in the pathophysiology of neurodegenerative diseases. In this regard, EFhd2 expression is increased in substantia nigra and was found associated with Lrrk2 in Parkinson’s disease (Meixner et al., 2011; Liscovitch and French, 2014). EFhd2 was also identified as associated with poly-GA C9Orf72 aggregates in amyotrophic lateral sclerosis and frontotemporal lobar degeneration, suggesting that EFhd2 may modulate the formation of pathological protein aggregates other than tau (May et al., 2014). Further characterization of EFhd2’s role in AD and other neurological disorders could lead to better understanding of the transition from physiological to pathological protein conformations that could serve as the basis for the identification of therapeutic strategies.

## Data Availability

The datasets generated for this study are available on request to the corresponding author.

## Author Contributions

IV did the experimental design, conducted the experiments, analyzed the data, and prepared the manuscript. AU performed the experiments, contributed to the data analysis, and proofread the manuscript. NK provided the purified recombinant tau protein, contributed to the data analysis, and proofread the manuscript.

## Conflict of Interest Statement

The authors declare that the research was conducted in the absence of any commercial or financial relationships that could be construed as a potential conflict of interest.
