# Supplementary figures and images for: EFhd2 Affects Tau Liquid–Liquid Phase Separation
Source: Front Neurosci. 2019 Aug 13;13:845. doi: 10.3389/fnins.2019.00845 (PMC6700279; doi:10.3389/fnins.2019.00845)

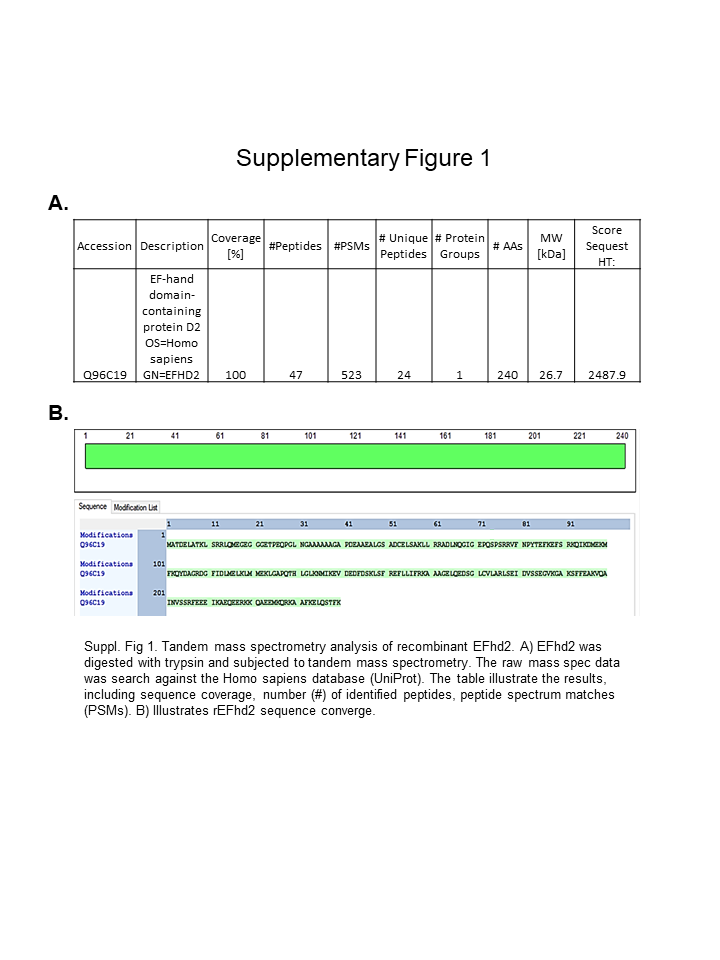

Supplement: Supplementary file 1 [file Image_1.TIF]

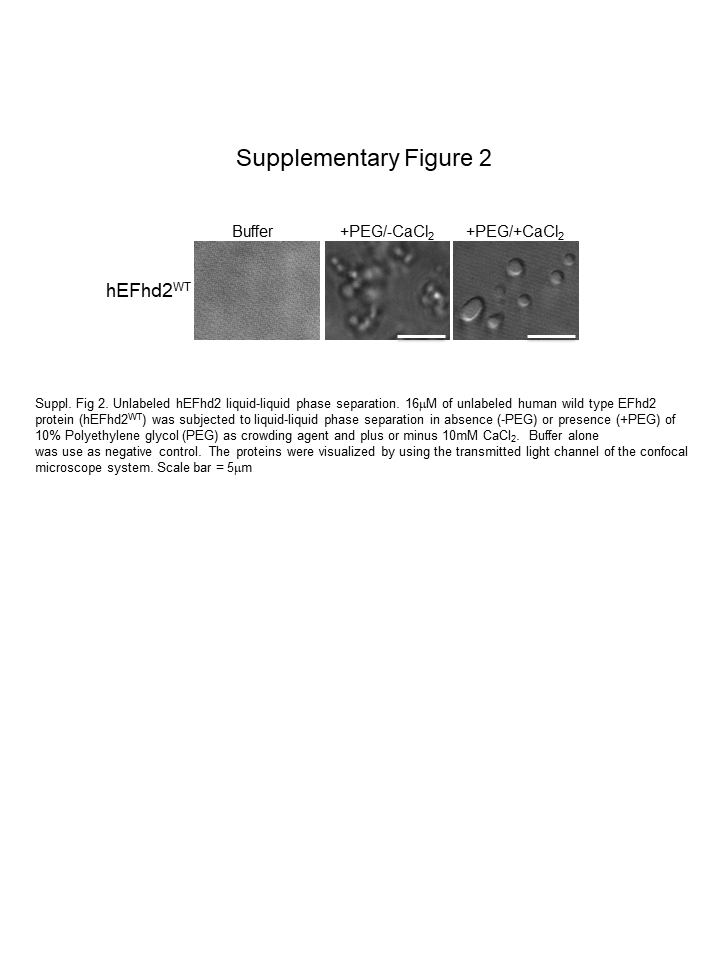

Supplement: Supplementary file 2 [file Image_2.TIF]

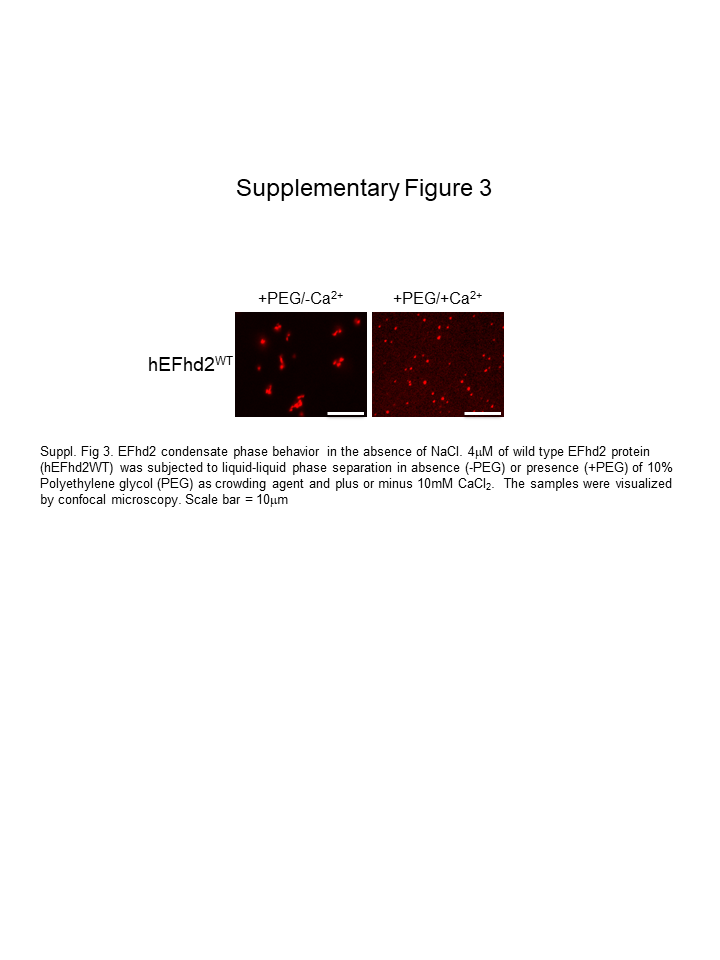

Supplement: Supplementary file 3 [file Image_3.TIF]

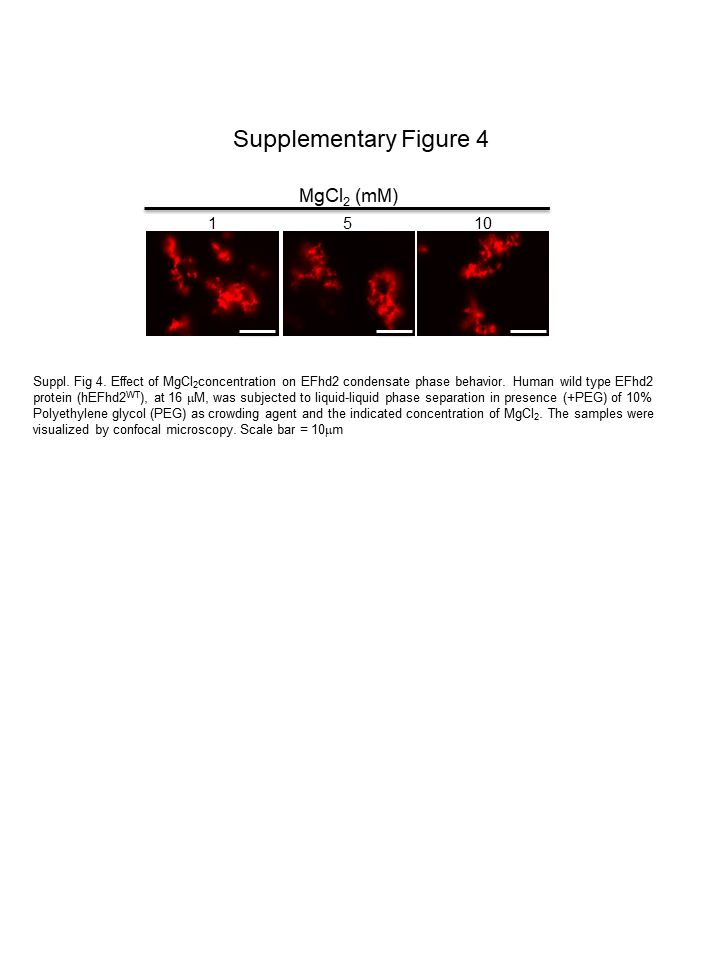

Supplement: Supplementary file 4 [file Image_4.TIF]

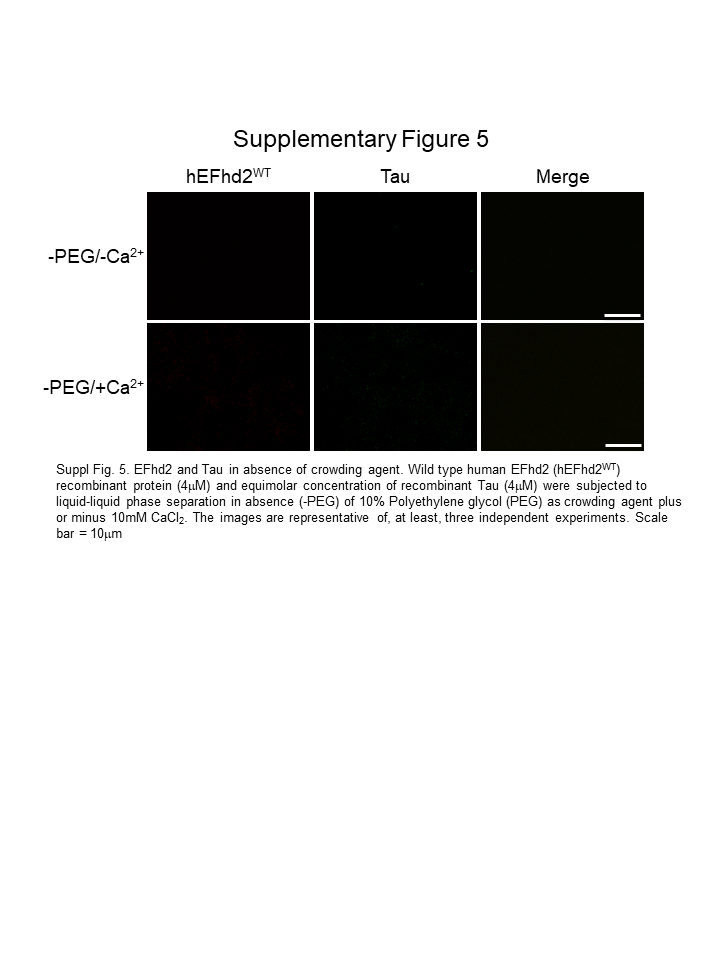

Supplement: Supplementary file 5 [file Image_5.TIF]
